# Polyneuropathy, Organomegaly, Endocrinopathy, M-protein, and Skin Changes (POEMS) Syndrome and Idiopathic Portal Hypertension: A Rare Association

**DOI:** 10.7759/cureus.24923

**Published:** 2022-05-11

**Authors:** Fatima Belabbes, Youssefi Houda, Abderahmane Al Bouzidi, Youssef Bennani, Maryame Ahnach

**Affiliations:** 1 Gastroenterology and Proctology, Faculty of Medicine, Mohammed VI University of Health Sciences (UM6SS), Casablanca, MAR; 2 Hematology, Faculty of Medicine, Mohammed VI University of Health Sciences (UM6SS), Casablanca, MAR; 3 Pathology, Faculty of Medicine, Mohammed VI University of Health Sciences (UM6SS), Casablanca, MAR

**Keywords:** liver biopsy, autologous stem cell transplants, idiopathic, portal hypertension, poems syndrome

## Abstract

Polyneuropathy, organomegaly, endocrinopathy, M-protein, and skin changes (POEMS) syndrome is a paraneoplastic syndrome due to an underlying plasma cell neoplasm. The diagnosis of POEMS syndrome is made with three of the major criteria, two of which must include polyradiculoneuropathy and clonal plasma (PCD), and at least one of the minor criteria. The most frequent liver manifestation is hepatomegaly. Idiopathic portal hypertension (IPH) has been reported rarely in POEMS syndrome. The precise etiopathogenesis of IPH is not fully elucidated.

We report a 46-year-old male patient presenting with POEMS syndrome. He presented postprandial vomiting and left tinnitus. Loss of appetite, leanness, and excessive sweat were concomitant symptoms. Abdominal examination revealed splenomegaly and an absence of hepatomegaly. Abdominal ultrasonography was therefore performed, revealing hepatosplenomegaly with dilatation of the splenic vein. An abdominal computed tomography confirmed the presence of an 18.5 cm splenomegaly with dilatation of the splenic and portal vein. Upper endoscopy with biopsy showed minimal, non-atrophic, mildly active chronic, follicular antrofundic gastritis without esophageal varices. Laboratory and radiological examinations could not confirm the etiology of portal hypertension. The liver biopsy suggested hepatoportal sclerosis, compatible with IPH. The patient initially received six courses of the CTD (cyclophosphamide, thalidomide, and dexamethasone) protocol. He subsequently proceeded to an autologous stem cell transplant (ASCT), and the patient achieved a considerable improvement.

POEMS syndrome could be complicated with IPH. There are only a few cases of IPH associated with POEMS syndrome in the literature. This case highlights the manifestation of portal hypertension in POEMS syndrome.

## Introduction

Polyneuropathy, organomegaly, endocrinopathy, M-protein, and skin changes (POEMS) syndrome is a rare hematological disorder caused by a plasma cell proliferation disorder. First coined by Bardwick in 1980, the diagnosis is currently made according to the updated Dispenzieri diagnostic criteria, requiring the presence of both mandatory criteria (polyradiculoneuropathy and monoclonal plasma cell-proliferative disorder) jointly with at least one major and one minor criterion [1]. The major criteria include the presence of sclerotic bone lesions, elevated serum levels of vascular endothelial growth factor (VEGF), and Castleman disease. The minor criteria encompass organomegaly, extravascular volume overload, endocrinopathy, skin changes, papilledema, and thrombocytosis or polycythemia. Idiopathic portal hypertension (IPH) is an extremely uncommon manifestation of POEMS syndrome.

Idiopathic non-cirrhotic portal hypertension (INCPH), however, is a rare disorder consisting of an increased portal venous pressure gradient in the absence of a known intrinsic cause of liver disease and portal vein thrombosis. To date, its pathophysiological mechanisms remain largely unknown, with a wide array of nosologic entities highly dependent on histological features and sharing several clinical elements [1]. We report an uncommon case of POEMS syndrome associated with INCPH. To the best of our knowledge, this article presents the first published clinical case of a patient with POEMS syndrome with IPH in our country.

## Case presentation

A 46-year-old man was initially admitted to our hospital with a prior three-year history of numbness in the lower and upper limbs, followed by asthenia and weakness resulting in difficulty in walking and standing. Several months prior to admission, his symptoms became severe, accompanied by cutaneous hyperpigmentation, postprandial vomiting, and left tinnitus. Loss of appetite, leanness, and excessive sweat were concomitant symptoms. Aside from his arterial hypertension, the patient had no other significant personal or family history. He was not exposed to several medications and toxins.

Upon general examination, he was conscious with an overall Glasgow Coma Scale score of 15, eupneic, normotensive, and apyretic. Physical examination was notable for cachexia, bilateral jugular carotid, and right inguinal adenopathy with splenomegaly. No pallor in mucous membranes was noted. Cutaneous examination revealed hyperpigmentation, particularly in the gingiva, buccal mucosa, and hard palate, along with achromic spots on the back. Bichat's fat pad atrophy, bilateral gynecomastia, pearly-white nails, acrocyanosis, and sclerodactyly were also noted.

Abdominal examination revealed only splenomegaly. Hepatomegaly, abdominal distension, and dilation of collateral veins over the anterior abdominal wall were absent. The first neurological assessment revealed muscle strength in his upper limbs, right lower limb, and left lower limb of grades of 4, 4, and 0, respectively. His physiological reflexes were normal and pathological reflexes were not elicited. Most notably, he had bilateral extremity numbness, particularly in the lower limbs, along with distal wasting. The proprioceptive sensitivity was intact, but the patient had a motor deficit in his lower limbs, unabling him to walk with a tendon retraction in all four limbs. The remaining systemic examination was normal, including cardiovascular and pulmonary examinations.

The electromyography revealed severe sensory-motor polyneuroradiculoneuropathy with predominantly axonal involvement in the lower limbs. The craniofacial computed tomography (CT) was normal. Moreover, the ophthalmological examination revealed papilledema staged 1. Initial laboratory investigations portrayed a normal blood count and liver biochemical tests. Serum creatinine and 24-hour urine total protein were elevated at 16.27 mg/L and 0.530 g, respectively. The other laboratory parameters are tabulated in Table 1.

**Table 1:** Laboratory data for our patient

Parameter	Normal laboratory value	Value
Hemoglobin, g/dL	13.0-18.0	13.2
White blood cells, per mm3	4,000-11,000	8,340
Number of platelets, per μL	150,000-400,000	348,000
Prothrombin time, %	70-100	87
Partial thromboplastin time	<1.2	1.2
Fibrinogen	2-4.5	4.41
International normalized ratio	0.8-1.2	1.10
C-reactive protein	<8	8.70
Procalcitonin	<0.5	1.28
Total bilirubin, mg/L	2-12	4.10
Unconjugated bilirubin, mg/L	2-7	2.50
Conjugated bilirubin, mg/L	<5	1.60
Alkaline phosphatase, IU/L	40-150	96
Aspartate aminotransferase, IU/L	5-34	10
Alanine aminotransferase, IU/L	<55	<6.00
γ-glutamyltransferase, IU/L	<85	36
Lactate dehydrogenase, IU/L	85-230	214
Urea, g/L	0.15-0.45	0.59
Creatinine, mg/L	7-13	16.27
24-hour urine total protein, g/24-hour	<0.15	0.530
Uric acid, mg/L	26-72	112.70
Albumin, g/L	34-50	36.28

**Figure 1:**
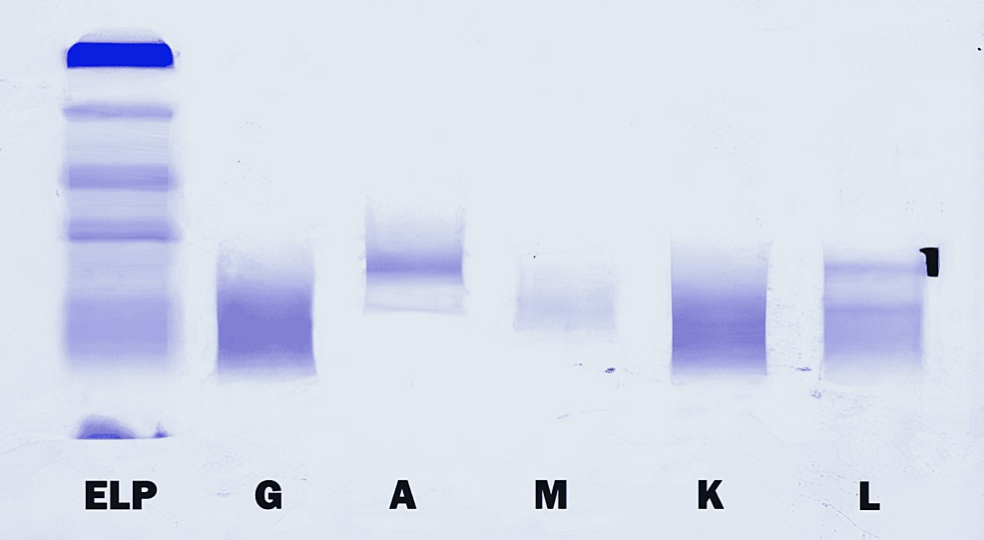
Urine protein electrophoresis showing absence of Bence Jones protein

Further, blood tests showed an elevated serum VEGF level. Serum protein electrophoresis and immunofixation revealed a monoclonal peak of the gamma-globulin area consisting of a monoclonal band of the IgA lambda type. There was no Bence Jones proteinuria in urine protein electrophoresis (Figure 1).

The karyotype was normal. Endocrine tests confirmed subclinical hypothyroidism, with elevated thyroid-stimulating hormone (TSH). He had low testosterone levels and normal blood sugar. A peripheral blood smear was normal and bone marrow aspirate and biopsy showed plasma cell dystrophy without a bone marrow plasmacytosis accompanied by cytological stigmata of a bone marrow reaction. Given the patient was presented with polyneuropathy, monoclonal gammopathy, elevated VEGF, organomegaly, endocrinopathy, and hyperpigmentation, the diagnosis of POEMS syndrome was established.

**Figure 2:**
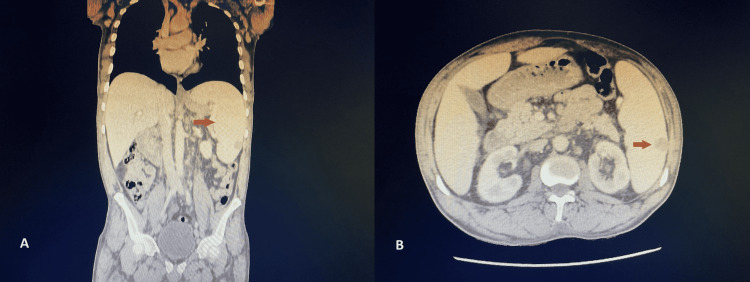
Abdominal computed tomography findings (A) Splenomegaly (red arrow) measuring 18 cm in diameter in coronal section. (B) Hypodense splenic nodule (red arrow) in cross-section.

Abdominal ultrasonography was therefore performed, revealing hepatosplenomegaly and splenic vein diameter at 12 mm. Portal vein diameter measured 6 mm and flow was hepatopetal. Neither nodular contour of the liver was revealed. An abdominal CT confirmed the presence of an 18.5 cm splenomegaly, splenic nodule, dilated splenic vein, supra, and subdiaphragmatic lymphadenopathy (Figure 2).

**Figure 3:**
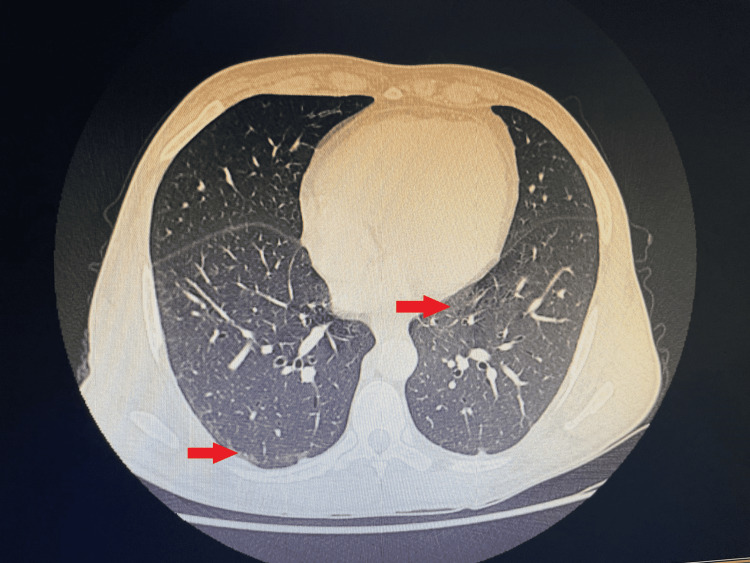
Thoracic computed tomography showing bilateral interstitial (red arrows) lung disease in cross-section

The portal vein was dilated at 17 mm, and there was no portal thrombosis. Minimal ascites between the intestinal loops were found. It did not detect signs of cirrhosis. The Doppler of the supra-hepatic veins was without abnormalities. A thoracic CT showed bilateral interstitial lung disease (Figure 3).

**Figure 4:**
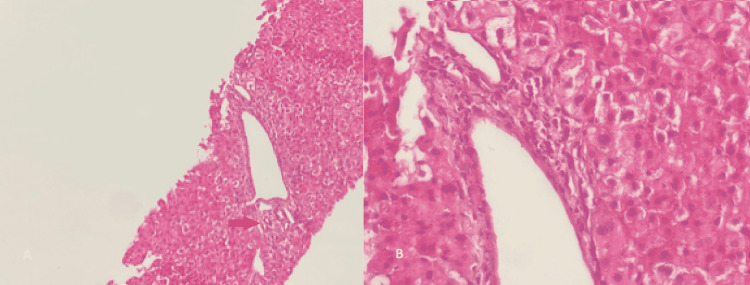
Liver parenchymal (A) Liver parenchymal showing enlarged portal spaces with minimal fibrosis associated with an inflammatory infiltrate (red arrow) (H&E, Gx50). (B) This inflammatory infiltrate is mainly made up of mononuclear elements with a predominance of lymphocytes (H&E, Gx400). H&E: hematoxylin and eosin stain; G: grossissement.

In addition, upper endoscopy with biopsy showed minimal, non-atrophic, mildly active chronic, follicular antrofundic gastritis without intestinal metaplasia or dysplasia. The absence of *Helicobacter pylori* and esogastric varices was also noted. Workup for viral hepatitis B and/or C, non-alcoholic or alcoholic steatohepatitis, autoimmune hepatitis, primary biliary cirrhosis, and prothrombotic were noncontributory. After obtaining the patient’s written informed consent, a liver biopsy was performed. The histological examination revealed chronic active hepatitis classified as activity 1 fibrosis 1 (A1F1) according to the Metavir grid, with no sign of cholestasis or any specific lesion or tumor compatible with IPH (Figure 4).

**Figure 5:**
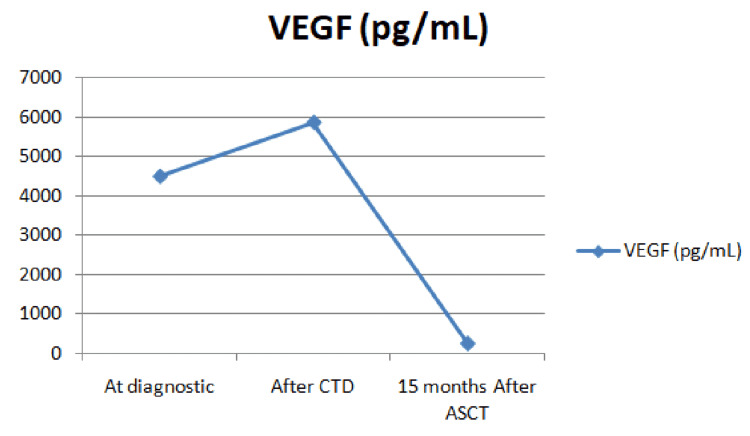
Serum VEGF values pre- and post-treatment with CTD or ASCT VEGF: vascular endothelial growth factor; CTD: cyclophosphamide, thalidomide, and dexamethasone; ASCT: autologous stem cell transplantation.

The diagnosis of POEMS associated with IPH syndrome was confirmed. The patient initially received six courses of the CTD (cyclophosphamide, thalidomide, and dexamethasone) protocol: oral intakes of cyclophosphamide 500 mg on days 1, 8, 15, and 22, 100 mg of thalidomide for 28 days continuously, and oral dexamethasone 40 mg on days one to four and 15 to 18. Supportive therapy consisted of hydration, allopurinol 300 mg, metoclopramide 10 mg, and antimicrobial and antithrombotic prophylaxis. He also received physical therapy and exercise as supportive care, along with replacement therapy for his endocrine disorder. The patient was under surveillance along with proton-pump inhibitors (PPIs). He subsequently proceeded to an autologous stem cell transplant (ASCT). The patient was therefore engrafted. The transplantation course was uneventful, and a follow-up examination revealed a significant neurological improvement. Numbness in lower limbs improved gradually, as for the sensory disturbance. Serum VEGF levels increased after CTD and were normalized 15 months after ASCT (Figure 5).

No vomiting or variceal bleed episodes have been noted. No jaundice or other gastrointestinal symptoms have been noted. A decrease in splenomegaly’s dimensions with no portal hypertension was also disclosed by abdominal ultrasound with the absence of hepatomegaly, ascites, and indirect signs of cirrhosis. The liver biochemical tests including cytolysis and cholestasis were within the normal range.

## Discussion

POEMS syndrome is a rare paraneoplastic disorder caused by an underlying plasma cell disorder. This syndrome includes a number of multi-system characteristic features, unrestricted to those included in the acronym. The disease remains rare and evidence for treatment is largely limited to retrospective cohort studies or case reports. Although POEMS has a worldwide distribution, it is particularly prevalent in France, the United States, China, and India [2]. The latter was initially thought to be more common in Japanese progeny, given the largest initial reports from Japan. Gender and age disparities have also been reported, with an onset median age in the sixth decade and a slight male preponderance, and overall survival of eight years. Though the underlying pathogenesis is yet to be determined, elevated levels of proinflammatory and angiogenic cytokines are constantly present in multi-organ involvement. Nevertheless, VEGF remains the cytokine that most correlates with the disease’s activity. Clinically, neurological features remain the hallmark of POEMS syndrome [3].

However, INCPH is a rare disorder consisting of an increased portal venous pressure gradient in the absence of a known intrinsic cause of liver disease and portal vein thrombosis. To date, its pathophysiological mechanisms remain largely unknown, with a wide array of nosologic entities highly dependent on histological features and sharing several clinical elements. INCPH diagnosis is based on a set of clinical criteria, and a formal exclusion of other causes of portal hypertension is mandatory. In this regard, liver biopsy is essential to rule out other causes of liver diseases, including cirrhosis. IPH is also world-widely distributed, with a median age at diagnosis of 40 years and predominance in the male gender.

As yet, there is scant literature regarding the association between POEMS and INCPH [4]. IPH’s etiopathogenesis is as yet not fully elucidated and uncommonly associated with POEMS syndrome. To date, their coexistence is scarcely reported in the literature [5]. Although it is still unclear whether IPH is part of the POEMS spectrum or develops independently, a hepatic circulation defect secondary to POEMS syndrome has been documented, involving pro-inflammatory cytokines such as VEGF, interleukin (IL)-6, IL-1ß, tumor necrosis factor-α, tumor growth factor-ß, and pro-thrombotic factors. The acquired IPH hypothesis incriminates several pathogenic factors including infections, drugs, toxic intake, and prothrombotic conditions.

Initially, the diagnosis of POEMS syndrome required the presence of three major criteria and at least one minor criterion. The major criteria were as follows: polyradiculopathy and monoclonal plasma cell proliferative disorder, which are mandatory, Castleman disease, sclerotic bone lesions, and VEGF elevation. The minor criteria included organomegaly, extravascular volume overload (such as edema, pleural effusion, and ascites), endocrinopathy (adrenal, thyroid, pituitary, gonadal, parathyroid, and pancreatic), skin changes (such as hyperpigmentation and hypertrichosis), papilledema, and thrombocytosis. Peripheral neuropathy remains the most predominant manifestation, as described in our patient’s case: peripheral, ascending, symmetrical, and affecting both motor and sensation functions. However, this marked clinical feature remains non-specific. This represents a clinical challenge, leading in many instances to misdiagnosing patients with diabetic peripheral neuropathy (DPN) [5].

INCPH has also been referred to as tropical splenomegaly, phlebosclerosis, portal veinopathy obliterans of the liver, hepatoportal sclerosis, non-cirrhotic portal fibrosis, and IPH [6]. Unlike POEMS syndrome, signs of INCPH may vary including splenomegaly, laboratory signs such as thrombocytopenia, or endoscopic signs of portal hypertension: esophageal varices but the liver function is usually preserved [7]. The clinical presentation can also be dominated by additional liver-related complications such as ascites, hepatic encephalopathy, portal vein thrombosis, or liver failure. The diagnosis of INCPH can only be made after the exclusion of other portal hypertension or non-portal hypertension causes. Therefore, detailed medical history for precipitating factors, namely, concomitant diseases, and drug or medication exposure is crucial. Liver function tests are generally within the normal range and may occur in the context of acute infectious episodes or variceal bleeding. Comprehensive liver imaging and biopsy are required to discard cirrhosis, Budd-Chiari syndrome, portal vein thrombosis, non-cirrhotic portal hypertension including chronic viral hepatitis, primary biliary cirrhosis, non-alcoholic steatohepatitis, alcoholic steatohepatitis, autoimmune hepatitis, and other systemic conditions causing portal hypertension such as sarcoidosis [8]. In our case, portal hypertension was probably associated with POEMS syndrome, since there are no investigations suggesting the presence of cirrhosis or other common causes of portal hypertension.

Several treatment modalities are recommended in POEMS ranging from localized to systemic protocols; however, there is scant literature regarding the evidence for different treatments as largely limited to retrospective cohort studies [9]. Treatments that have been tried include chemotherapy, radiation, corticosteroids, thalidomide, and blood stem cell transplant [7].

The prognosis of POEMS syndrome remains good after treatment. Favorable prognostic factors for overall survival include albumin greater than 3.2 g/dL, attainment of a complete hematologic response, and younger age, according to a report from the Mayo Clinic [7]. Moreover, patients with relapse also responded effectively to second-line treatments, and similarly, patients presenting several relapses to further treatment options. Nevertheless, a close follow-up remains essential. The overall survival of patients with POEMS syndrome has improved given the use of new therapies targeting directly the clonal plasma cell disorder rather than solely directing it at VEGF [9].

As for INCPH, there is a considerable lack of randomized controlled trials regarding its management strategy and prophylaxis of its complications, namely, variceal bleeding. Up to now, experts endorse opinions following the current recommendations for cirrhosis-related portal hypertension, as no guidelines for INCPH are yet established [10]. The use of pharmacological treatments including non-selective beta-blockers and endoscopic variceal ligation is deemed very effective for preventing variceal bleeding. For patients who fail to respond to the aforementioned therapies, transjugular intrahepatic portosystemic shunting (TIPSS) can be suggested. Management of an acute variceal bleeding episode includes the use of vasoactive drugs, concomitant endoscopic control of bleeding, blood product replacement, and prophylactic antibiotics. Guidelines also recommend early withdrawal of potential drugs associated with INCPH and treating any precipitating medical conditions [11]. Overall, the prognosis of patients with INCPH is generally favorable in contrast to patients with cirrhosis, mainly due to their preserved liver function.

## Conclusions

In summary, POEMS syndrome is a paraneoplastic syndrome due to an underlying plasma cell neoplasm. IPH is a rare disorder characterized by clinical portal hypertension in the absence of a recognizable cause such as cirrhosis. INCPH in POEMS syndrome has been described rarely in the literature. This case highlights the manifestation of portal hypertension in POEMS syndrome. It is not necessary to have all the signs of POEMS and IPH syndrome to make the diagnosis, knowing that this can only be conceived within a multidisciplinary framework. Early recognition of the two syndromes association is important to reduce morbidity.
